# From Sunlight to Screens: Modeling When Light Exposure Matters Most for Sleep and Circadian Health

**DOI:** 10.3390/clockssleep8020021

**Published:** 2026-04-27

**Authors:** Franco Tavella, Michael Gradisar, Renske Lok, Olivia Walch

**Affiliations:** 1 Arcascope Inc., 4075 Wilson Blvd, Floor 8, Arlington, VA 22203, USA; 2 Sleep Cycle AB, Sleep Science Team, 41250 Gothenburg, Sweden; 3 Department of Psychiatry and Behavioral Sciences, Stanford University, Stanford, CA 94305, USA; 4 Department of Neurology, University of Michigan, Ann Arbor, MI 48104, USA

**Keywords:** light exposure, sleep, circadian health

## Abstract

Understanding the effects of light on the body at different times of the 24 h solar day is a topic of increasing interest. In this paper, we use a mathematical model from the literature to simulate what would be expected of the human circadian clock on different light schedules. We first reproduce an influential experiment which found eBooks, when compared to a paper book, delayed sleep by roughly 10 min and melatonin onset by 1.5 h. The model is able to match the delay in sleep onset but struggles to reproduce the melatonin phase delay. However, certain initial conditions and parameters are capable of phase shifts consistent with the original study’s magnitude, suggesting that the original study’s finding may have been influenced by the pre-study entrainment or variability among the participants. We next simulate the same protocol under higher daytime light levels (increasing baseline illumination from 90 to 500 lux) and find that brighter daytime exposure reduces both sleep onset latency and the variability in phase delay attributable to evening eBook light. Finally, we explore how the timing of a bright light pulse during the day changes outcomes, such as sleep onset and circadian amplitude, and how these effects interact with light during the other hours of the 24 h day. Together, these modeling results suggest robust daytime light exposure confers resilience against the circadian-disruptive effects of evening light, generating testable predictions regarding the timing and intensity of beneficial light interventions for maintaining circadian alignment.

## 1. Introduction

People are increasingly interested in the effects of light on the human body. This trend is visible in both online search activity related to “light and health”, which has quintupled since 2012 [1], as well as in the rapidly growing markets for blue-blocking glasses and smart lighting systems, which are projected to expand substantially over the coming decade [2,3,4,5]. From a circadian research perspective, it is exciting to see a growing awareness of the many physiological effects that light has on the body. At the same time, there has been heightened focus on getting or avoiding light at specific times of the waking day, while ignoring other times of the day (e.g., “[T]here must be a cut-off time in the morning when it’s pointless to try to get your sunlight in”—Reddit user, from the post “How late into the morning can I still get sunlight and feel the benefits?” [6]).

Simple advice tends to resonate most, so it is no surprise (and not necessarily bad) that many social media recommendations boil down to “get bright light in the morning” and “avoid light before bed” [7,8]. However, seminal and emerging research data show these periods are not the only times light matters for sleep and circadian health. In humans, there is little evidence for a circadian “dead zone”—a period during which light has no effect on phase shifting—that is commonly observed in other mammals [9,10,11]. Rather, the phase response curves to light exhibit “dead points”, as phase advancing regions cross over to phase delaying regions and vice versa [12,13,14]. Moreover, the times when light least affects circadian phase may be when it most affects circadian amplitude, a challenging quantity to measure but one that, within an individual, can be taken to reflect the strength of the body’s circadian clock [15].

To add to the complexity, an individual’s recent lighting history significantly shapes how the eye—and, by extension, the brain [16]—responds to new light signals [16,17,18]. The retina’s sensitivity to light is not fixed; it adapts based on prior exposure, meaning that sleep and circadian responses to a given light stimulus in the evening can vary dramatically depending on the context of preceding light–dark patterns during the day. The magnitude and direction of light’s influence on sleep and circadian timing are modulated by recent lighting history—including light intensity, duration, spectral composition, and timing throughout the day and night [19,20,21,22]. Since light exposure at any one time interacts dynamically with the broader lighting context, the full-day lighting environment is crucial for predicting physiological and behavioral outcomes in response to an intervention given at a specific time.

Comprehensively investigating the effects of many possible light exposure patterns—characterized by differences in intensity, duration, and timing—on subsequent sleep and circadian parameters will require hundreds of hours of research time and millions of dollars in research funding. In contrast, mathematical modeling offers a fast and cost-effective approach to evaluate lighting paradigms and generate testable hypotheses. Relevant to this paper, modeling can help us understand the conditions under which morning and/or evening light is expected to have significant (or minimal) effects on the sleep and circadian system. These effects are captured in the model by the phase of the oscillator, amplitude of the oscillator, and the current saturation of the retina to light (collectively, the “circadian state”), as well as the amount of homeostatic pressure for sleep. Phase from the model can be used by itself to predict dim light melatonin onset (DLMO), the gold standard biomarker for the central circadian pacemaker [23], while phase and homeostatic pressure from the model can be used to predict downstream outputs like sleep onset [24]. Models moreover allow us to simulate combinations of lighting conditions and individual phenotypic parameters, such as light sensitivity [25,26]. This approach allows us to begin to address questions that require a scale and level of control unattainable (or at least extremely difficult) through experimental methods alone.

In this paper, we use a mathematical model to simulate how light exposure throughout the day shapes the response to morning and evening light [27]. Our simulations are based on Skeldon et al.’s [27] model which combines a limit cycle representation of the human circadian clock with a homeostatic sleep process. We identify an ensemble of parameter sets for this model that yield physiologically realistic sleep durations higher than 6.5 h (see Section 4). This setup allows us to mirror typical experimental conditions and jointly analyze circadian and sleep outcomes across a range of biologically plausible parameter sets.

We use this model to reproduce a highly influential study, Chang et al. (2015) [28], assessing the effects of evening eBook light on sleep latency and circadian phase. In that study, participants were kept in a lab under 90 lux conditions during the day and exposed to either an eBook (30 lux) or a paper book (in 3 lux conditions) from 6:00 p.m. to 10:00 p.m. daily before providing a circadian assessment with DLMO on the 7th day. Participants then switched to the other condition and repeated the same exposures. Whether the eBook or paper book was presented first was randomized by subject. That study found a 1.5 h phase shift and a 10 min increase in sleep onset latency under the eBook condition when compared to a paper book.

In addition to simulating using the same daytime lux values reported in the original study—90 lux—we also simulate the case where daytime lux value is brighter (500 lux). We selected 500 lux as a representative daytime indoor light level because it approximates commonly recommended illumination levels for indoor environments such as offices, classrooms, and healthcare settings [7]. For example, lighting guidelines and empirical measurements of typical indoor environments often report daytime illuminance levels in the range of ~300–500 lux at eye level [7]. Building on these results, we then simulate the effects of brief light pulses at different times of the day, against a range of different baseline lux values, to examine how these exposures influence circadian phase and amplitude as well as sleep-related outcomes. Such simulations can assist our interpretations and translations of laboratory experiments to real-world situations [22].

## 2. Results

### 2.1. eBook Simulations

**Figure 1:**
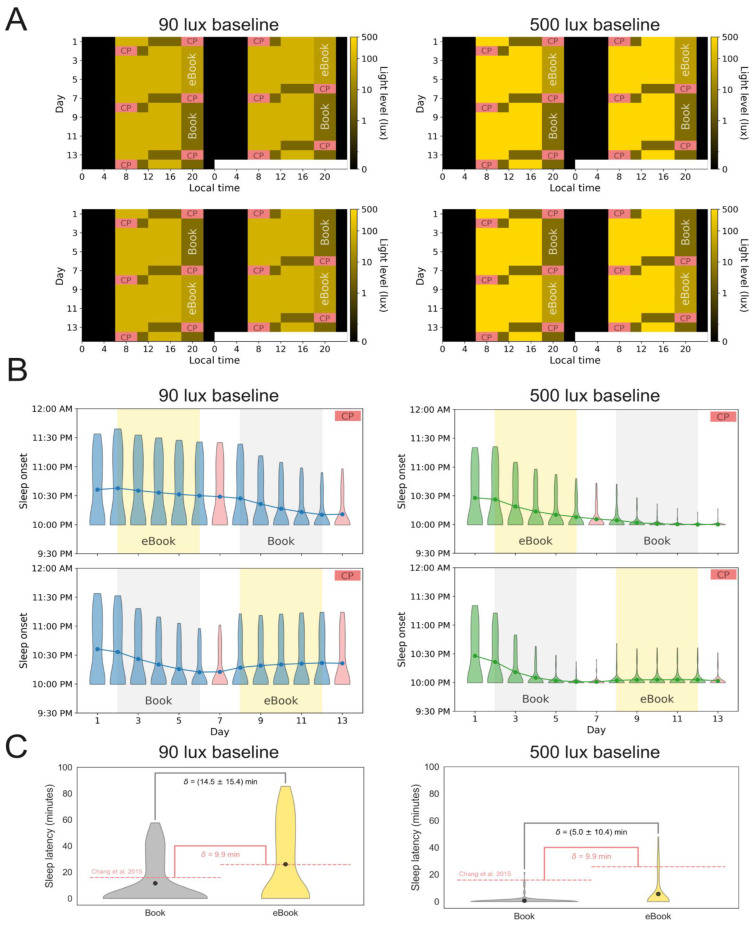
(**A**) The experimental protocol from Chang et al. [28] (left) as well as our modified version for simulations. Digitized schedules from Chang et al. 2015 [28] under two different baseline lux conditions (left: 90 lux, right: 500 lux) are shown. Simulations were performed for the two possible reading condition sequences (top: eBook-first week, bottom: eBook-second week). Yellow brightness corresponds to the relative light brightnesses in the protocol, with darkest yellows corresponding to the dimmest lights. Pink regions correspond to periods of melatonin collection under the constant posture (CP) procedures. (**B**) Time evolution of sleep onset for subjects with different sleep parameters over the course of the simulated protocol shown in A. The distribution is represented by a violin plot and mean values by circle markers. Constant procedure days are denoted as red violin plots. Left: 90 lux baseline (blue), right: 500 lux baseline (green). Top: eBook-first week, bottom: eBook-second week. (**C**) Sleep latency distribution for each reading condition with 90 lux baseline simulations on the left and 500 lux baseline on the right. Mean sleep latency is denoted as black dots. The mean sleep latency difference between eBook (gray) and Book (yellow) conditions is denoted by *δ*, plus or minus its standard deviation. Values reported in Chang et al. [28] for the fourth and fifth nights of each condition are shown in red (15.75 min for book condition and 25.65 min for eBook condition; 9.9 min difference).

Sleep onsets and latencies. Figure 1A provides a schematic overview of the laboratory conditions under various lighting and experimental conditions, including (i) the real daytime lab lighting conditions of 90 lux from the Chang et al. [28] study (left panels) and a simulated daytime lighting condition of 500 lux (right panels), and (ii) the eBook–Book sequence across the 2-week lab study (top panels) and the reversed Book–eBook sequence (bottom panels). This four-panel schematic (Figure 1A) aligns with the modeling of sleep onset times (Figure 1B), with Figure 1C demonstrating the differences in sleep onset latencies (SOL) in the 90 vs. 500 lux conditions. Here, the mathematical model reproduces the modest increase in SOL observed in Chang et al. (2015) [28], with a 14.5 min average delay under 90 lux daytime lighting in the mathematically simulated SOL compared to the 10 min the researchers reported [28] (Figure 1C, left panel). When simulated under 500 lux daylight (Figure 1C, right panel), SOL decreases to 5 min, which aligns with most previous studies [29].

**Figure 2:**
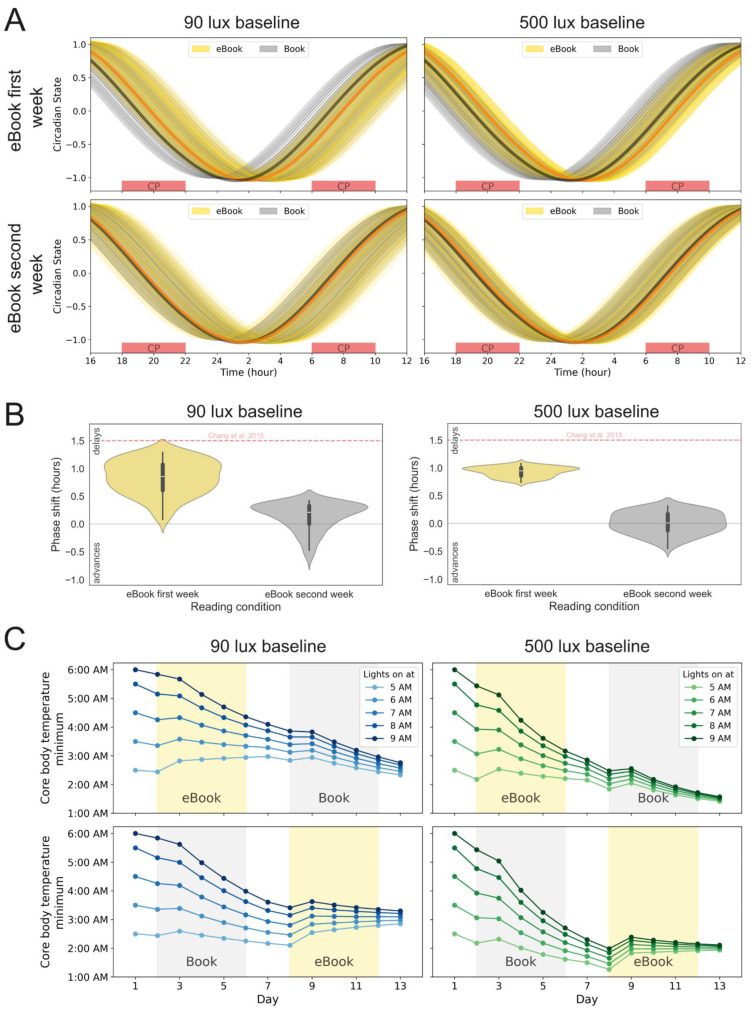
(**A**) Time evolution of circadian phase and amplitude, expressed through the variable *x* in [24,27], where *x* is assumed to hit its lowest value at CBTmin, for subjects with different circadian parameters on the last day of each reading condition (yellow: eBook, gray: Book). Thick lines represent the behavior of the model default parameters (orange: eBook, black: Book). Left: 90 lux baseline, right: 500 lux baseline. Top: eBook-first week, bottom: eBook-second week. Experimental constant procedure times (melatonin collection) are denoted as red boxes. (**B**) Phase shift grouped by reading condition order. Left: 90 lux baseline light, right: 500 lux baseline light. White lines represent the median of the distribution. The red dashed line indicates the 1.5 h shift reported in Chang et al. [28] (**C**) Core body temperature minimum evolution for different equilibration light schedules. Left: 90 lux baseline (blue), right: 500 lux baseline (green). Top: eBook-first week, bottom: eBook-second week. Colors correspond to different initial conditions, with darker lines corresponding to later behaviors prior to the study.

Circadian timing differences. Chang et al. [28] originally reported a 1.5 h phase delay between the two studied conditions, with rhythms shifted 1.5 h later on average in the eBook condition than in the paper book condition. In our simulations, we calculate phase shifts as differences in simulated core body temperature minimum timing between constant posture days. Our simulations are only able to reproduce the 1.5 h phase difference between the two conditions when the eBook is provided first (eBook–Book, Figure 2A,B, left panels) and only for a limited set of parameters (near the top of the violin plot, see Table S1 in Supplementary Material). Indeed, the likelihood of a 1.5 h phase delay appears about as frequently as a small advance in the eBook-first condition (Figure 2B, left, see endpoints of the yellow violin plot). The rarity of achieving a 1.5 h phase delay for the eBook relative to the paper book in the simulations holds even as light sensitivity and other circadian parameters are varied (Figures S3–S5).

The order of presentation (eBook–Book vs. Book–eBook) influences the extent of the phase shift observed. Under the 90 lux condition we find a median of 51.3 min phase delay observed for the eBook-first condition and a median of 12.6 min phase delay observed for the eBook-second condition in the mathematical simulations (Figure 2B, left panel). For the 500 lux conditions those values are a 56.7 min phase delay for the eBook-first condition and a 0.6 min phase delay for the eBook-second condition.

The fact that order matters in determining the amount of phase shift observed may be due to the fact that the individuals, assumed to be entrained to a 10:00 p.m. lights-off time (per the methodology in [28]), are being phase advanced by both the paper book and eBook conditions relative to their starting phase before the trial. In other words, while a phase delay may appear to have occurred when comparing phase after the eBook exposure to phase after the paper book exposure, the outcome under both conditions could be advanced relative to the individuals’ starting phase.

This can be understood as follows: the protocol has individuals in 3 lux of ambient room lighting in the paper book condition (Figure 1 in [28]) and roughly 30 lux in the eBook condition (from the eBook) between 6:00 p.m. and 10:00 p.m., which is likely much darker than their habitual lighting during those hours under free-living conditions [30,31]. Thus, they experienced a dark pulse during the phase delay region, which would act to advance participants. If the eBook phase advanced participants less than the paper book, then it would appear as a phase delay between the two conditions.

More importantly, if a participant is starting from a very different light schedule than the one in the study protocol, their pre-study habitual light schedule could influence the magnitude of the phase advance experienced under these dark pulses as well as the observed phase difference between the eBook and paper book conditions. Trajectories of CBTmin over time for different initial conditions (i.e., habitual lights-on times before study start) are shown in Figure 2C. The simulations in Figure 2A,B assume a 6:00 a.m. lights-on time as the starting point, consistent with the original protocol in [28]. In Figure 2C, in contrast, the changes in phase that would be expected over the course of the trial for a range of pre-study lights-on times (from 5:00 a.m. to 9:00 a.m.) are shown.

When the eBook is given first (under the study conditions of 90 daytime lux), all trajectories except the one corresponding to a pre-study lights-on time of 5:00 a.m. experience a phase advance between days 1 and 7, which is followed by all trajectories phase advancing under the paper book condition between days 7 and 13 (Figure 2C, top left). When the eBook is given second, there is first a large phase advance across all trajectories under the paper book condition, which then regresses slightly and stabilizes under the eBook condition (Figure 2C, bottom left).

The end result is that the modeling predicts a larger difference between eBook and paper book when the eBook is used first, versus when the paper book is used first, in ways that depend on the schedule the participant was entrained to prior to the trial. While the individuals in the Chang et al. study [28] were assumed to be entrained to a 6:00 a.m. lights-on schedule (10:00 p.m. lights off), an individual entrained to a 9:00 a.m. lights-on time and put into the eBook-first condition would appear to have been delayed 1.34 h by the eBook relative to the paper book. That same person, however, would appear to have been advanced 0.31 h by the eBook had they been under the paper book condition first (Figure 2C, left, dark blue curve on days 7 and 13).

Ninety lux, even when measured at the ocular level, is very dim relative to the light available outdoors and through windows [31]. When the same simulations are repeated with 500 lux as the baseline (Figure 1A, right), versus the 90 lux in the original experiment [28], the effects observed are reduced, with a 5 min delay in sleep onset observed when averaged across all parameter sets (Figure 1B,C, right). The phase delay is similarly reduced, with an average phase shift of approximately 1 h when the eBook is provided first and close to no phase shift observed in the paper-book-first (Book–eBook) condition (Figure 2A,B, right). Trends in the role of initial conditions, with phase advances occurring to different extents over the course of the simulated study, remain consistent (Figure 2C).

### 2.2. Pulse and Stability Simulations

**Figure 3:**
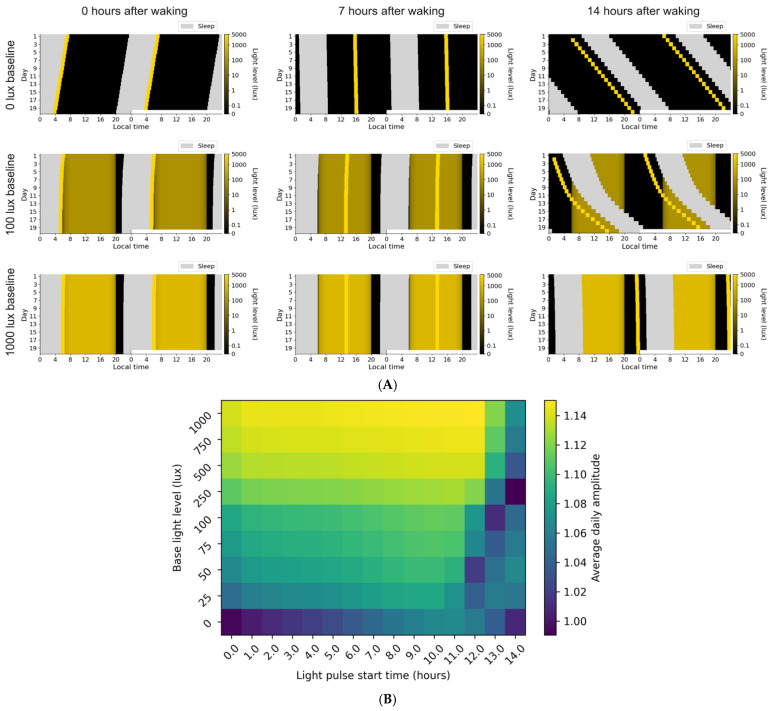
(**A**) Actograms showing the effect of a pulse of light for different levels of baseline light. The vertical axis shows day of simulation, while the horizontal axis shows time of day (double plotted). The brightness of the light provided to the simulation at that time is shown in the colorbar. Sleep is shown as gray regions (during which photic input to the circadian clock is assumed to be zero). Across simulations, light linearly increases from 0 to a baseline lux value between 6:00 a.m. and 8:00 a.m. and linearly decreases back to zero from 6:00 p.m. to 8:00 p.m. The top row corresponds to a baseline lux value of 0; the middle corresponds to 100 lux as the baseline, and the bottom corresponds to 1000 lux. In all simulations, a 5000 lux pulse of light is given *N* hours after waking, where *N* is 0, 7, or 14. Top to bottom: 0, 100, 1000 baseline lux. Left to right: Pulse at 0, 7, 14 h after waking, to capture a phase advance, an amplitude boost, and a phase delay. (**B**) Heatmap of average circadian amplitude for a 1 h pulse of 5000 lux at different hours after waking (horizontal axis) and different values of base light levels (daily maximum lux value, vertical axis).

Our simulations of the eBook study show how the daytime light level can influence observed outcomes, with brighter daytime light leading to decreased sleep onset latency from eBook light exposure and reduced influence of interindividual variability on circadian phase. To more broadly examine how daytime light levels interact with pulses of light at different times of day to influence sleep behaviors, simulations were conducted using fixed baseline levels of ambient light (Figure 3A) to which a 5000 lux pulse was applied at different times.

When pulses of bright light are the only light source (i.e., baseline lux level of zero), bright light upon waking causes a phase advance and an observed circadian period length shorter than 24 h (indicating that the phase advance exceeds the 0.2 h daily adjustment required for a 24.2 h intrinsic period). In contrast, high-intensity light exposure 7 h after waking produces minimal phase change, while exposure 14 h after waking produces a significant phase delay and an observed circadian period longer than 24 h (Figure 3A, upper row). Entrainment occurs when the baseline lux is 100 or 1000 lux (Figure 3A, middle and bottom row) and the light pulse is given 0 and 7 h after waking. In all cases where a 5000 lux pulse is given 14 h after waking (Figure 3A, last column), a drift at a period greater than 24 h is seen, but the speed of this drift is reduced when the baseline light is higher.

When the baseline is 100 lux, bedtime in the “light immediately upon waking” simulations (Figure 3A, first column) occurs earlier than the 7 h after waking simulations (Figure 3A, middle column) by approximately 52 min, while in the 1000 lux baseline case, bedtime occurs approximately 21 min earlier. In both cases, total sleep time is nearly identical within a baseline lux condition. In the 100 lux baseline, there are 7:23 h of sleep when the pulse occurs at 7 h. In the 1000 lux baseline, total sleep times are 7:31 and 7:32 for the 0 and 7 h after waking pulse times, respectively.

While phase shifts and circadian period are important markers of circadian dynamics, amplitude also plays a critical role in determining the strength and stability of circadian rhythms, as discussed in [15]. To understand the effects of pulse timing and baseline light on model-derived circadian amplitude, baseline light levels ranging from 0 to 1000 lux and pulse times ranging from 0 to 14 h after waking were evaluated. Average circadian amplitude was calculated by averaging across the entire last day of simulation. A brighter baseline lux tends to increase circadian amplitude, so long as the pulses occur during the middle of the waking hours (Figure 3B). Maximal circadian amplitude was found on the 1000 lux baseline schedules (the brightest tested), with pulses approximately 5–11 h after waking, while lowest circadian amplitude was found on schedules where the baseline lux was lowest and the pulses happened at the extremes of the day (either immediately after waking or close to bed).

## 3. Discussion

Modeling can help interpret outcomes of past experiments and generate new, testable hypotheses with minimal overhead cost. In doing so, we can examine potential mechanisms by which lighting history and circadian state influence the effect of light at a given moment in time. For most, light exposure in the morning is going to phase advance the circadian clock [32,33] while light in the evening will delay it [34,35,36,37]. The absence of light during a period when it is habitually experienced would be expected to have the opposite effect, with an absence of habitual light in the morning producing a phase delay (relative to the previously entrained schedule) and the absence of habitual light in the evening producing a phase advance.

Using a model based on Skeldon et al.’s work [27], we first simulated an eBook study [28] in which individuals entrained to a 6:00 a.m. wake time were kept in 90 lux during the day and exposed to either dim light conditions of 3 lux while reading a paper book or 30 lux from an eBook from 6:00 p.m. to 10:00 p.m. The model is readily able to reproduce a similar sleep onset latency difference between the two conditions, akin to that reported in the original experiment. However, the modeling rarely reproduces the 1.5 h phase delay attributed to the eBook in the original work despite the many parameter sets assessed.

There are two possible explanations for the inability of the mathematical model to match the observed phase shift in our work. First, because the modeling expects that the protocol was phase advancing for both the eBook and paper book conditions, a larger phase shift between the conditions could have been observed if the participants were entrained to a later schedule prior to study start than the assumed 10:00 p.m. lights-off time. A person entrained to a later habitual schedule before coming into the lab and assigned to the eBook–Book condition could appear later at the end of the eBook condition, not due to eBook light phase shifting them later but rather the fact that their prior schedule was markedly different from the study protocol schedule (and they had not finished entraining to the study schedule at the time of the phase assessment). Second, while the average phase shift deviated substantially from the values reported by Chang and colleagues [28], there were some parameter combinations that did reach a 1.5 h phase delay, suggesting that the effect observed could have been driven by individuals with phenotypes loosely corresponding to those parameters (see Figure S5 for the distributions as intrinsic period, light sensitivity, and phase response curve shape are changed). That said, the likelihood of observing a 1.5 h delay in the eBook-first condition with the parameter ranges tested was roughly the same as that of observing a small phase advance (Figure 2B, left), suggesting that the same experiment, repeated again, could yield very different results if inter-individual differences were the cause.

In [28], the authors made a significant effort to control for initial conditions in their participant pool, noting “Participants were also required to maintain a fixed 8-h sleep schedule (10:00 PM to 6:00 AM), to complete a daily sleep/wake log, and to call in their bedtimes and wake times every day during this 3-wk interval. This sleep schedule was verified by wrist actigraphy (Actiwatch-L; Philips/Respironics) during the week before admission” (p. 1235). From this, it appears unlikely that a participant entrained to a later schedule would not have been identified and excluded from participation. At the same time, our own experience in human subject trials and actigraphy has led us to believe that anything is possible outside the lab, with devices sometimes purposefully left off or transferred to another person when real-life demands collide with the requests of the research team. As such, it is not inconceivable that a person entrained to a later schedule could have made it past the authors’ rigorous efforts to control for this variable.

Had the baseline light during the day in [28] been of higher intensity, our modeling suggests that the difference in observed sleep onset latency would have been reduced (from 14.5 min to 5 min). The magnitude of this effect is consistent with results from a recent meta-analysis summarizing multiple studies on screen use at night [29]. Similarly, the maximum circadian phase delay seen across parameter sets is reduced when the baseline lux is increased in our simulations, though a delay is still reliably observed in the eBook-first condition. The higher-intensity daytime lux condition reduced the spread in the amount of observed phase shift by more quickly entraining participants to the study conditions than the 90 lux protocol. Future protocols could place participants on highly entraining schedules (very bright light during the day, total darkness at night) at the beginning of or throughout an experimental protocol to reduce the effects of initial conditions and inter-individual differences on the outcomes of the study.

In addition to simulating experimental protocols, modeling can also make predictions for experiments which have not been conducted. For instance, models can make testable hypotheses around how the broader temporal pattern of light exposure—beyond a discrete pulse of bright light—influences the effects of the pulse, through both clock state (circadian phase and amplitude) as well as retinal saturation (captured as Process L in the models used). In general, higher-intensity light during the daytime period, over a duration comparable to typical daily sunlight, had a stabilizing effect on the sleep–wake system, with pulses having less ability to shift sleep/wake timing when baseline light exposure was brighter. While the system was usually able to entrain to pulses of light delivered 0 and 7 h after waking, it was unable to entrain to bright light pulses 14 h after waking, with the extent to which drift occurred mitigated by the baseline daytime light level.

Higher-intensity light during the day reduced the impact of a morning light pulse on sleep timing in our modeling. When baseline light was 1000 lux, light upon awakening and 7 h after waking produced similar effects on circadian clock phase timing and sleep onset times, with the light pulse 7 h after waking (middle of the day) resulting in sleep schedules that were on average 21 min later than the schedules where light was given immediately upon waking. This difference was further amplified when baseline light was reduced to 100 lux. Under this condition, a light pulse delivered 7 h after waking delayed sleep onset by 52 min compared with a pulse delivered immediately upon waking. Light delivered during the middle of the day had a larger boosting effect on circadian amplitude than light early in the morning or late in the day, consistent with predictions from previous modeling [15].

In all cases, more baseline light acted as a stabilizer, with higher-intensity light during the day dramatically reducing the impact of a light pulse 14 h after waking. Depending on an individual’s phenotype (e.g., light sensitivity or intrinsic circadian period) and intervention goals (e.g., shifting circadian timing versus strengthening rhythmicity), circadian amplitude may in some cases represent a more suitable target than circadian phase. Consistent exposure to higher-intensity daytime light is one of the clearest strategies for increasing circadian amplitude [15].

It is critical to note that all of our results are based on mathematical models which themselves are limited representations of physiology. The retinal processing in the model used is based on photopic illuminance, with an initial higher transient response followed by a lower steady state response. Full retinal modeling would include melanopic irradiance and distinct models for the contributions of rods, cones, and intrinsically photosensitive ganglion cells. We largely simulate light as constant levels or pulses, when real-world light can be sporadic and spectrally diverse. Moreover, there are non-circadian effects of light on the body that are beyond the scope of these models. Our models similarly do not account for the alerting effects of light, which can be valuable in the morning. We also report outcomes for circadian amplitude, which is at present more of a theoretical construct than a measure with an agreed upon, easy-to-capture gold standard [15]. Finally, our models do not include the dynamics of melatonin in the circadian/sleep–wake system, with predicted DLMO simply derived from the phase state variable. As such, outcome variables such as melatonin suppression are outside the scope of our analyses.

Yet models exist to make hypotheses. The simulations presented here, generated from groundbreaking models developed by several of the authors of [28] and in use by a number of research groups around the world, suggest that phase shifting effects from eBooks are likely to be significantly reduced when initial state is controlled for and when baseline light during the daytime is made brighter. They also suggest that light immediately after waking and/or light in the middle of the day produce very similar outcomes, so long as the baseline light level is sufficiently high. This implies that there is no cut-off point in the morning past which light exposure is irrelevant. Rather, circadian health is a product of light across the whole 24 h period. The optimal advice may not be to “get light in the morning and avoid phones at night” but rather to seek the brightest days and the darkest nights while strongly maintaining inter-day lighting regularity. Future work should examine how interventions which target light across the full waking day, along with darkness across the whole night, compare to interventions that focus only on morning light or evening light avoidance.

## 4. Materials and Methods

The light schedule from Chang et al. (2015) [28] was digitized for use in our simulations (Figure 1A, left). Briefly, in that protocol, participants were in 90 lux during the daytime period, given an eBook or a paper book (depending on which group they were assigned to) to read between the hours of 6:00 p.m. and 10:00 p.m. in 3 lux light, and then allowed to sleep from 10:00 p.m. to 6:00 a.m. After five days in one condition (eBook or paper book), participants were switched to the other condition the following week. On the last day of the condition, as well as the day after, plasma melatonin samples were collected. These were used to determine both the extent of melatonin suppression (on the last day of the condition) as well as the extent of circadian phase shift (the day after).

A limit cycle model of the human circadian clock, which uses light history as its input, was used to predict circadian phase, circadian amplitude, and retinal saturation, or the circadian state, over time. The circadian clock is modeled as a van der Pol oscillator with an intrinsic period near 24 h, driven by light inputs via a photoreceptor saturation process; light reaching the retina shifts the phase and speed of the oscillator in a manner consistent with empirical phase response curves. The sleep process follows a two-threshold model in which homeostatic sleep pressure rises during wake and decays during sleep, with sleep onset and offset occurring when pressure crosses circadian-modulated upper and lower thresholds, respectively. Phase was used to predict timing of core body temperature minimum (CBTmin), which was assumed to happen 7 h after DLMO [38]. We only include results from one model [27] in this manuscript. However, future work on the open-source circadian package (https://github.com/Arcascope/circadian) would allow the expansion of these two-process results to the other circadian frameworks already present as standalone models in our package [39,40,41,42].

Sleep modeling was performed using a recent implementation of the two-process model [24,27]. Sleep was prevented in the presence of light above a threshold (0.5 lux) to mimic the study conditions, in which participant wake times were prescribed during set daytime hours. As a number of model parameters can yield similar sleep phenotypes and the forced wake up by light imposes new restrictions, a hyperparameter search was conducted to understand how different parameter choices influenced our outcomes. The key parameters varied were upper threshold of sleep pressure (μ), difference between upper and lower sleep pressure thresholds (Δ), and sleep pressure decay rate (ꭓ). The final ranges chosen for these parameters were μ ∈ [17.5, 19.5], Δ ∈ [5,9], and ꭓ ∈ [7,11] since they yielded sleep durations longer than 6.5 h and switched between sleep/wake only twice per day. We simulated a total of 125 parameter sets. All parameter sets identified that yielded at least 6.5 h of sleep (n = 61/125, 48%) were included.

Additional modeling of circadian parameters was performed by varying the parameters light sensitivity (p), phase response curve shape (*κ*), and intrinsic circadian period (*τ*_C_) in the circadian model. The ranges chosen for these parameters were p ∈ [0.5, 0.7], *κ* ∈ [0.4, 0.7], and *τ*_C_ ∈ [23.8, 24.4]. A total of 125 light sensitivity parameter sets were simulated. For all sets, sleep parameters were μ = 19.0, Δ = 6.0, and ꭓ = 11.0. All parameter sets yielded at least 6.5 h of sleep. Figure S2 shows the range of parameters assessed and full details of the modeling can be found at https://github.com/ftavella/evening_light_article (accessed on 1 December 2025).

For the simulations of the eBook-vs-book protocol [28], each of these parameter sets was simulated under two light schedules as per the Chang et al. [28]) protocol: (1) the condition with the eBook in the first week and paper book in the second week, and (2) the condition with the eBook in the second week and paper book in the first week. In all cases, parameter sets were equilibrated for 30 days on a regular schedule (constant 1000 lux from 6:00 a.m. to 10:00 p.m.) prior to the eBook simulation, unless otherwise specified, in order to match the protocol described in Chang et al. (2015) [28].

To simulate the effects of a bright light pulse during the day in the presence of a baseline background light, the same implementation of the two-process model was used [27]. For each simulation, light was set to a nighttime lux value before 6:00 a.m. and after 10:00 p.m. with a linear ramp to the brighter base light level being evaluated between 6:00 a.m. and 8:00 a.m., as well as a linear ramp to the nighttime value from 8:00 p.m. to 10:00 p.m. For all simulations except the base lux of 0 lux, the nighttime value was assumed to be 50 lux; for the full darkness case, the base lux was assumed to be 0. During sleep, 0 lux was used as input to the circadian model. Simulations were run for 20 days and the last day was used for analysis. A range of base light levels and pulse times after waking were evaluated, and average amplitude in the model was extracted from the simulations for analysis. In addition, actograms were produced to show whether entrainment occurred for 1 h pulses of 5000 lux occurring immediately upon waking, 7 h after waking, and 14 h after waking, for base lux values of 0, 100, and 1000 lux.

## 5. Conclusions

Modeling can illuminate how light over the 24 h day affects health. We find that modeling reproduces experimental work showing subtle differences in light exposure in the evening leading to subtle differences in sleep onset latency. We show that large phase delays observed from eBook light relative to paper books are possible for some parameter sets but that an alternative explanation for the magnitude of the phase delay is that individuals were not fully entrained to a 10:00 p.m. lights-off prior to study start. By simulating light pulses at different times of the day, we show that greater light during the waking period makes the sleep–wake system more robust to pulses of bright light across the day and that bright light pulses immediately after waking and during the middle of the day can be argued to have distinct positive outcomes (shifting sleep earlier vs. enhancing circadian amplitude). This modeling produces testable hypotheses that can be evaluated in future work.

## Data Availability

The original data and code presented in the study are openly available at https://github.com/ftavella/evening_light_article (accessed on 1 December 2025).

## Supplementary Materials

The following supporting information can be downloaded at: https://www.mdpi.com/article/10.3390/clockssleep8020021/s1.
