# Supplementary material for: From Sunlight to Screens: Modeling When Light Exposure Matters Most for Sleep and Circadian Health
Source: Clocks Sleep. 2026 Apr 27;8(2):21. doi: 10.3390/clockssleep8020021 (PMC13214838; doi:10.3390/clockssleep8020021)
Supplement: Supplementary file 1 [file clockssleep-08-00021-s001.zip › clockssleep-4039419-supplementary.pdf]

clocks & sleep

MDPI

Supplemental Materials

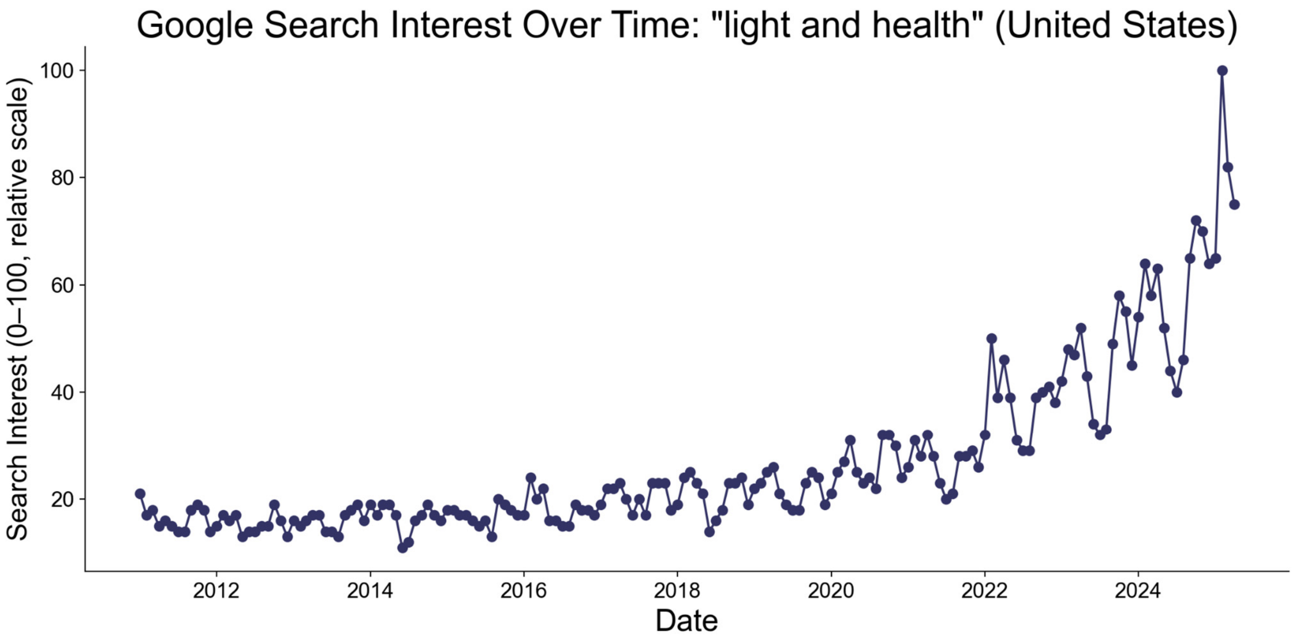

Figure S1. Interest in the search term “light and sleep” in the United States on Google, starting from January 1, 2011. Search interest is normalized by the number of total search queries and reflects growing interest in the topic, not growing usage of Google.

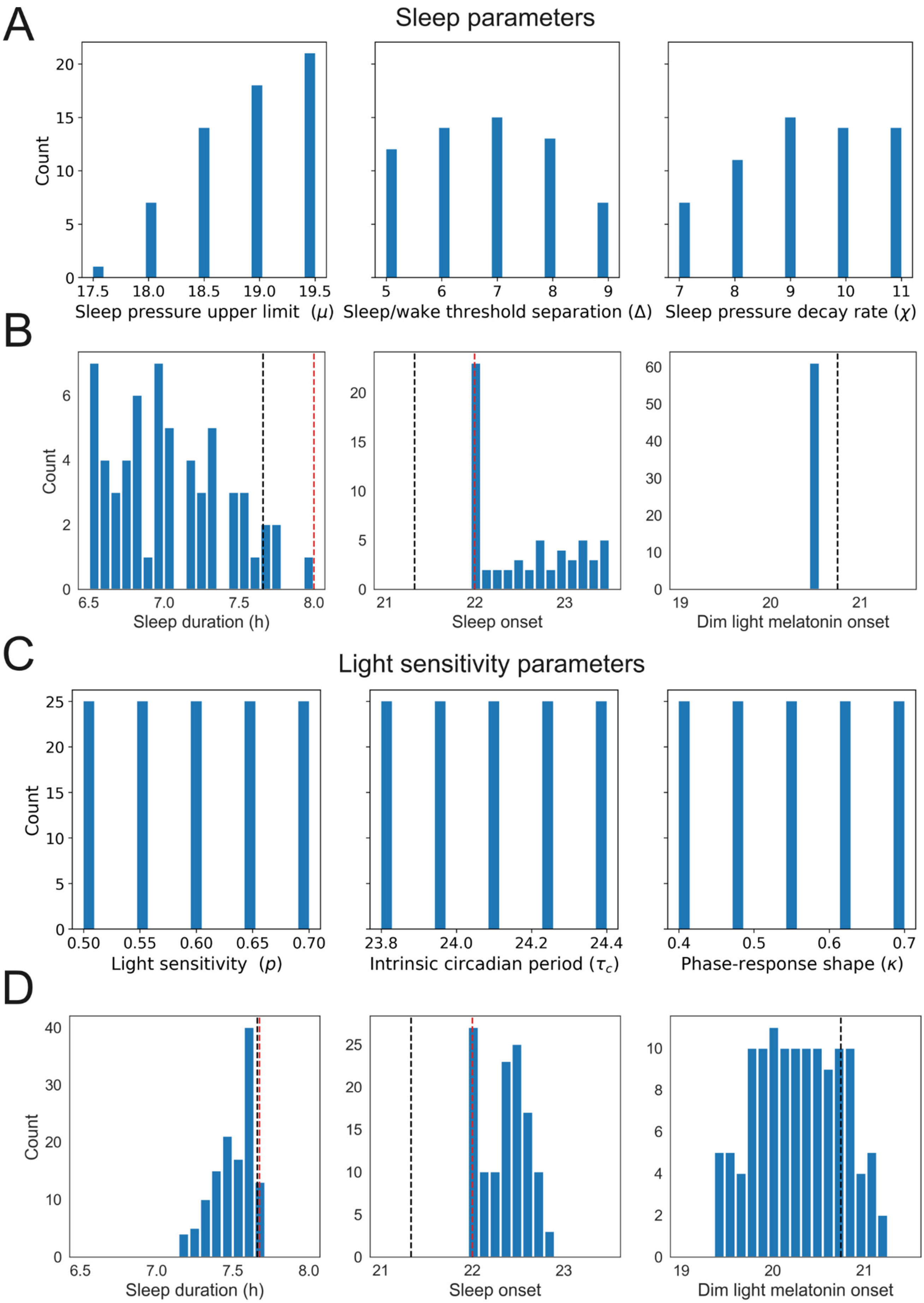
A
Sleep parameters
Count
Sleep pressure upper limit (μ)
Sleep/wake threshold separation (Δ)
Sleep pressure decay rate (χ)
B
Count
Sleep duration (h)
Sleep onset
Dim light melatonin onset
C
Light sensitivity parameters
Count
Light sensitivity (p)
Intrinsic circadian period (τ_c)
Phase-response shape (κ)
D
Count
Sleep duration (h)
Sleep onset
Dim light melatonin onset

Figure S2: Distributions of sampled sleep and circadian parameters. (A) Distribution of simulated sleep parameters with sleep duration of at least 6.5 hours. From left to right: upper limit of sleep pressure (μ), sleep/wake threshold separation (Δ), and sleep pressure decay rate (χ). (B) Distribution of sleep metrics for simulated sleep parameters (left: sleep duration, center: sleep onset, right: dim light melatonin onset). Black dashed vertical lines indicate the resulting metrics without forced wake up by light. In sleep duration and onset, the red dashed vertical line indicates the longest sleep duration and earliest sleep onset due to forced wake up by light. (C) Distribution of simulated circadian parameters. Since all tested parameters had a sleep duration of at least 6.5 hours, the distributions are uniform. From left to right: light sensitivity (p), intrinsic circadian period (τ_C), and phase-response curve shape (κ). (D) Distribution of sleep metrics as in (B) for simulated circadian parameters.Vertical black dashed lines indicate results without forced wake up by light and red vertical dashed lines indicate maximum sleep duration and minimum sleep onset due to forced wake up by light.

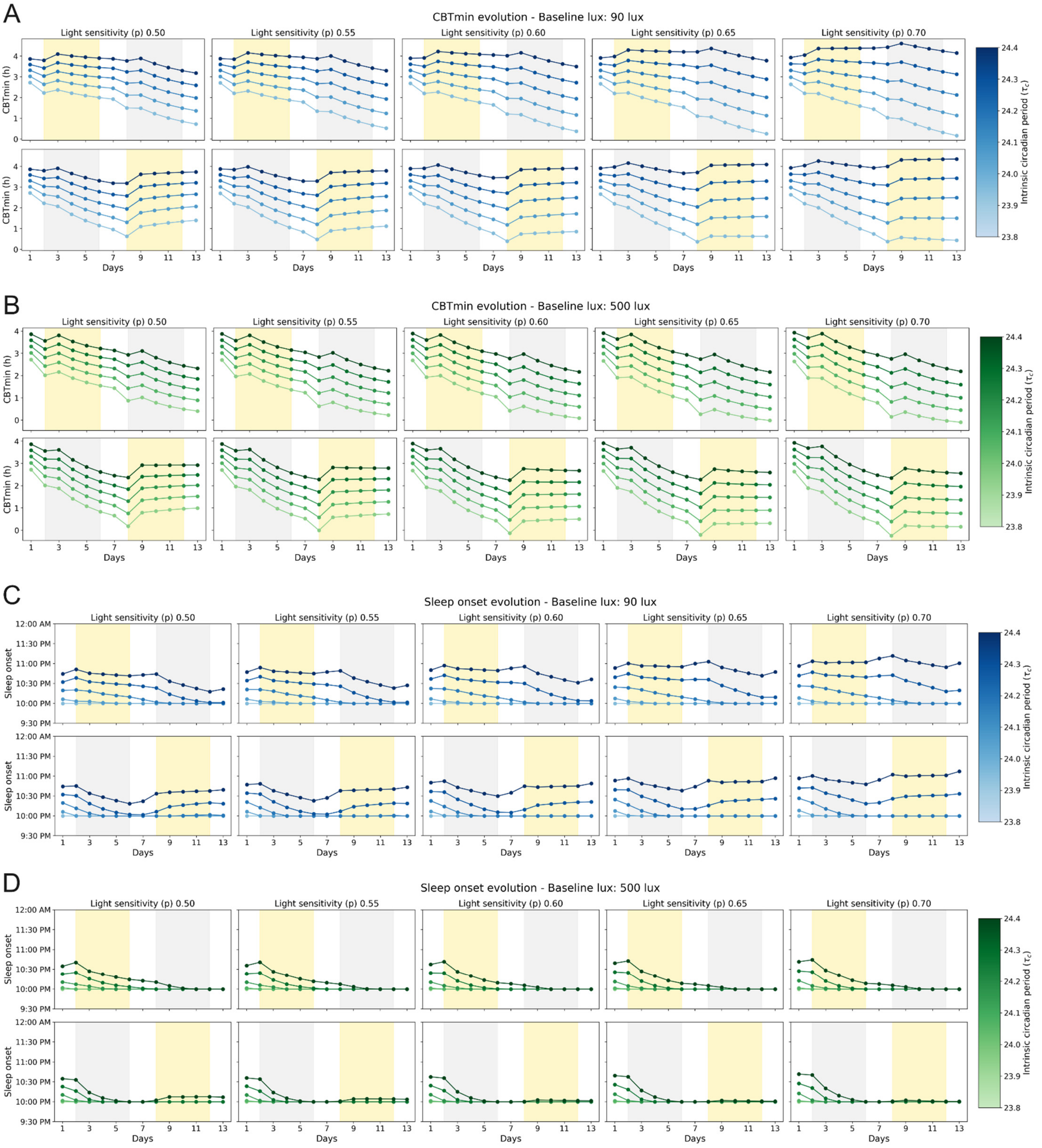

Figure S3: CBTmin and sleep onset over time for differing baseline lux levels and parameter choices. (A) Evolution of CBTmin over the days for the 90 lux baseline condition. (B) Same as A but for the 500 lux baseline condition. (C) Sleep onset evolution for the 90 lux baseline condition. (D) Same as C but for the 500 lux baseline condition. In all plots, data is grouped from left to right by light sensitivity and colored by intrinsic circadian period.

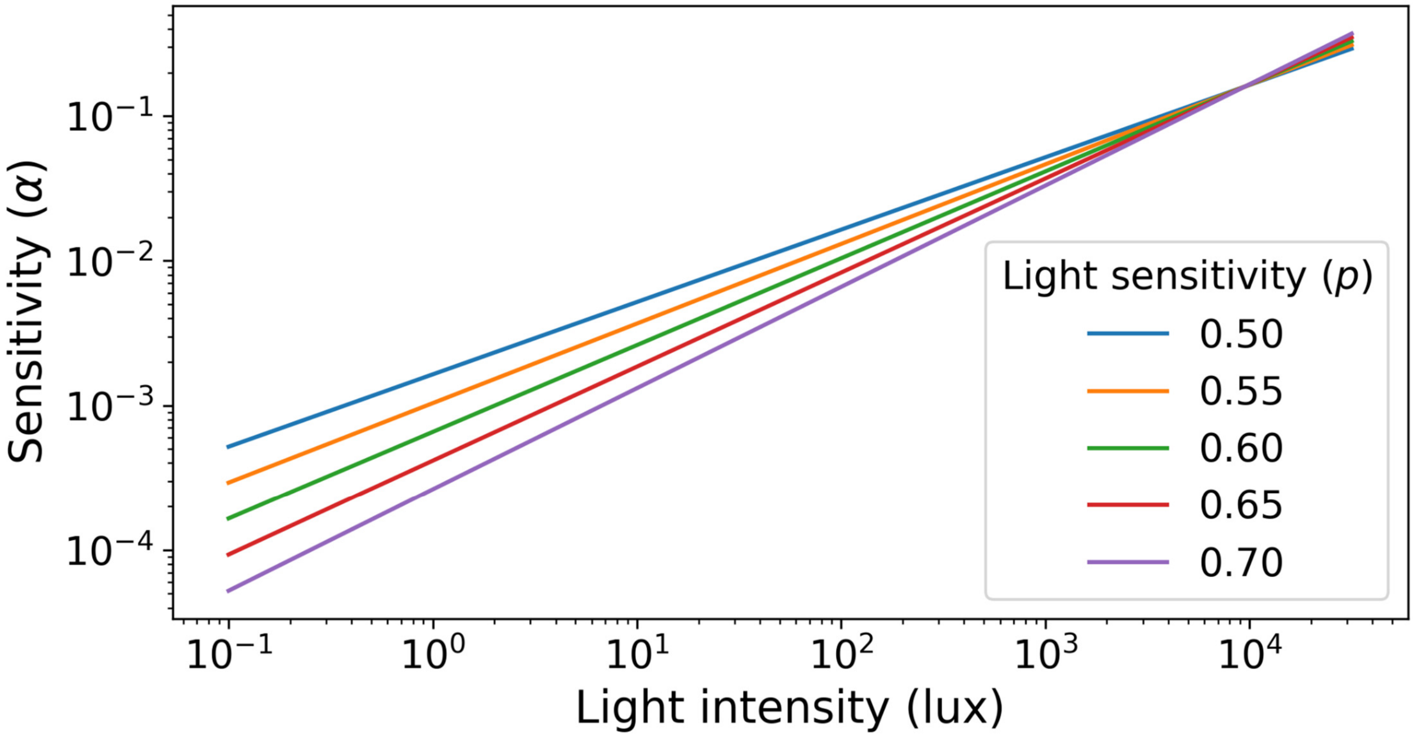

Figure S4: Intrinsic light sensitivity as a function of input lux for different values of the parameter p.

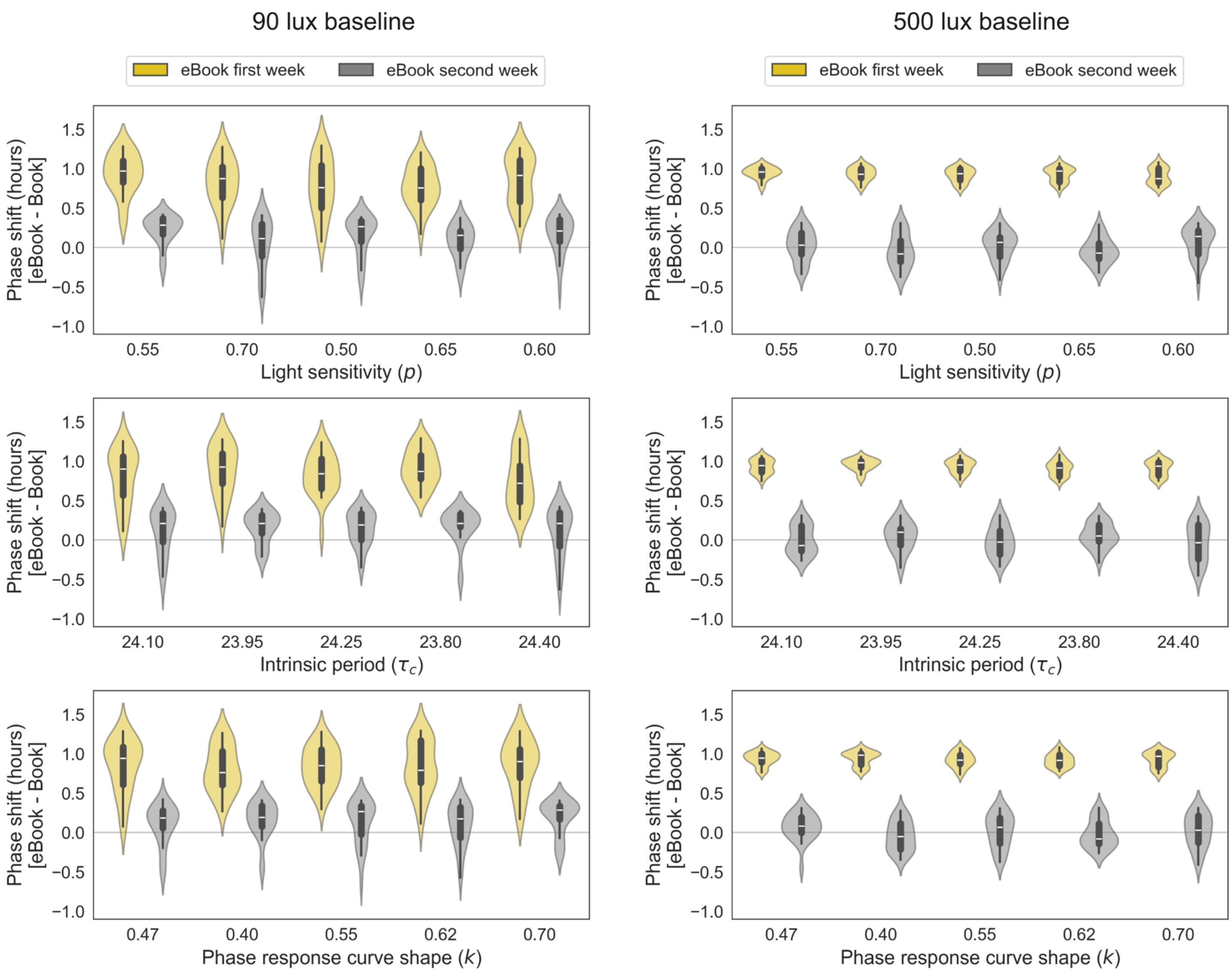

Figure S5: Phase shift for different values of circadian parameters (90 lux baseline left, 500 lux baseline right). From top to bottom, phase shift as a function of light sensitivity, intrinsic period, and phase response curve shape, respectively. Values are grouped by reading condition order.

Table S1: Maximum and minimum phase shifts for each condition and their corresponding parameters.

Baseline lux	Condition	Max/Min Shift (h)	Parameters
90	eBook First	1.29	$p = 0.6,\ \kappa = 0.625,\ \tau_c = 24.25$
		0.075	$p = 0.6,\ \kappa = 0.4,\ \tau_c = 24.1$
90	eBook Second	0.42	$p = 0.7,\ \kappa = 0.4,\ \tau_c = 24.4$
		-0.63	$p = 0.55,\ \kappa = 0.55,\ \tau_c = 24.4$
500	eBook First	1.08	$p = 0.7,\ \kappa = 0.625,\ \tau_c = 24.25$
		0.73	$p = 0.65,\ \kappa = 0.55,\ \tau_c = 24.25$
500	eBook Second	0.31	$p = 0.5,\ \kappa = 0.625,\ \tau_c = 24.1$
		-0.45	$p = 0.7,\ \kappa = 0.4,\ \tau_c = 24.4$
